# Systematic Identification of mRNAs Recruited to Argonaute 2 by Specific microRNAs and Corresponding Changes in Transcript Abundance

**DOI:** 10.1371/journal.pone.0002126

**Published:** 2008-05-07

**Authors:** David G. Hendrickson, Daniel J. Hogan, Daniel Herschlag, James E. Ferrell, Patrick O. Brown

**Affiliations:** 1 Department of Chemical and Systems Biology, Stanford University School of Medicine, Stanford, California, United States of America; 2 Department of Biochemistry, Stanford University School of Medicine, Palo Alto, California, United States of America; 3 Howard Hughes Medical Institute, Stanford University School of Medicine, Palo Alto, California, United States of America; Wellcome Trust Sanger Institute, United Kingdom

## Abstract

microRNAs (miRNAs) are small non-coding RNAs that regulate mRNA stability and translation through the action of the RNAi-induced silencing complex (RISC). Our current understanding of miRNA function is inferred largely from studies of the effects of miRNAs on steady-state mRNA levels and from seed match conservation and context in putative targets. Here we have taken a more direct approach to these issues by comprehensively assessing the miRNAs and mRNAs that are physically associated with Argonaute 2 (Ago2), which is a core RISC component. We transfected HEK293T cells with epitope-tagged Ago2, immunopurified Ago2 together with any associated miRNAs and mRNAs, and quantitatively determined the levels of these RNAs by microarray analyses. We found that Ago2 immunopurified samples contained a representative repertoire of the cell's miRNAs and a select subset of the cell's total mRNAs. Transfection of the miRNAs miR-1 and miR-124 caused significant changes in the association of scores of mRNAs with Ago2. The mRNAs whose association with Ago2 increased upon miRNA expression were much more likely to contain specific miRNA seed matches and to have their overall mRNA levels decrease in response to the miRNA transfection than expected by chance. Hundreds of mRNAs were recruited to Ago2 by each miRNA via seed sequences in 3′-untranslated regions and coding sequences and a few mRNAs appear to be targeted via seed sequences in 5′-untranslated regions. Microarray analysis of Ago2 immunopurified samples provides a simple, direct method for experimentally identifying the targets of miRNAs and for elucidating roles of miRNAs in cellular regulation.

## Introduction

MicroRNAs (miRNAs) are ∼22 nucleotide non-coding RNAs that regulate protein production by pairing to appropriate complementary stretches in mRNAs [1]–[4]. Hundreds of miRNAs are encoded in the human genome, with an estimated 30% of mRNAs possessing conserved miRNA binding sites, suggesting that miRNA-based regulation is an integral component of the global gene expression program [5], [6]. The importance and functional range of miRNAs is evident from their widespread occurrence and the diverse and often strong phenotypes and disease states associated with mutation or altered expression of miRNAs [7]–[14]. miRNAs function through formation of a ribonucleoprotein complex termed the RNA-induced silencing complex (RISC) [2], [15]–[17]. In humans, RISC is minimally composed of a guide miRNA bound to an Argonaute protein (Ago 1, 2, 3 or 4), along with Dicer and the HIV transactivating response binding protein (TRBP) [16]–[24]. Experiments in mice and human cell lines show that Ago2 is the central RISC component, capable of cleaving target mRNA when there is perfect miRNA:mRNA complementarity [21], [24]–[29]. However, most miRNA:mRNA interactions in metazoans have imperfect complementarity [30], [31], and it is likely that an overwhelming majority of miRNA targets are not cleaved by Ago2. In many cases it is likely that miRNAs repress translation and/or promote decay of their mRNA targets [11], [32]–[47].

A combination of experimental and computational approaches has begun to elucidate how mRNA targets are specifically recognized by miRNAs. From this large body of work, several salient features of target recognition have emerged. First, it is likely that most miRNA target sites are located in 3′-untranslated regions (UTRs) of mRNAs [6], [30], [31], [46], [48]–[52]. Sites in coding sequences and, in at least one instance, 5′-UTR can also lead to decreased protein levels, although they do so less efficiently than sites in 3′-UTRs [6], [43], [50], [53]–[55]. Second, a stretch of six to eight nucleotides near the 5′-end of the miRNA, the “seed region”, are particularly important for miRNA function [30], [31], [43], [48]. Their importance is underscored by the fact that the complementary regions are among the most evolutionarily conserved regions in mRNA targets and in some instances the seed match alone appears sufficient to confer recognition [6], [30], [31], [52], [56].

The observation that miRNAs cause decreases in the abundance of at least some mRNA targets provides a powerful strategy for determining what features in mRNA and miRNA sequences contribute to specificity [11], [33], [34], [36], [37], [39], [40], [42], [43], [45]. Recently, Lim *et al.* found that transfection of each of two miRNAs, heart-specific miR-1 and brain/kidney-specific miR-124, into HeLa cells led to decreases in abundance of at least 96 and 174 mRNAs respectively, many of which were likely to be direct targets as inferred from the enrichment of seed matches in their 3′-UTRs (∼90% had 6mer seed matches) [43]. The observation that many of these targets had conserved seed matches in their 3′-UTRs and that overexpression of the miRNA induced a muscle-like or brain-like gene expression program, respectively, suggested many of the apparent targets were physiological, even though miR-1 and miR-124 are not normally present in HeLa cells. In addition to the 3′-UTR sites, the authors found evidence for some targeting to sites in coding sequences. This miRNA overexpression/microarray approach was subsequently expanded to 11 miRNAs and used to identify additional features in mRNAs that contribute to changes in target mRNA levels [50]. These data provided the basis for a model for the effectiveness of each seed match site in 3′-UTRs of mRNAs for ∼450 miRNAs (TargetScan 4.0). Other miRNA target prediction methods are based on limited experimental data and theoretical considerations (*e.g.* mRNA secondary structure surrounding predicted sites), but only limited functional data are available to test their performance [31], [51], [52], [57]–[59].

One limitation of current approaches is that targets are often inferred from changes in mRNA abundance; however, miRNA-induced decreases in protein levels can only partially be accounted for by changes in mRNA levels, consistent with the view that miRNAs affect both translation and mRNA decay [11], [32]–[47]. In addition, identifying targets by altering miRNA expression and measuring changes in mRNA levels returns no information on which targets might be the most important in carrying out the actual biological processes (*e.g.* cellular differentiation) and is limited to the study of the altered miRNA. Conservation is commonly used as a filter to identify likely targets, but many functional sites are not conserved and many conserved sites do not seem to be functional [50], [60]. Although useful, the existing methods may be capturing an incomplete and possibly biased subset of miRNA targets.

A direct experimental method to identify miRNA targets that does not rely on any specific mechanism of regulation, conservation, or the altered expression of specific miRNAs is required to fully explore the suite of miRNA targets. Here we describe a simple method that provides quantitative information about which mRNAs are being regulated by miRNAs in a cell population. We express affinity-tagged Ago2 in Human Embryonic Kidney (HEK) 293T cells, immunopurify the resulting tagged Ago2 complexes, and identify the associated mRNAs and miRNAs using DNA microarrays. This Ago2 immunopurification (IP)/microarray approach allows miRNA targets to be comprehensively identified in an unbiased fashion, and provides a method for comprehensively assessing the regulation of mRNAs by RISC. In addition, mRNA targets of particular miRNAs can be identified by comparing the Ago2 IP/microarray profiles of cells expressing a particular miRNA to the Ago2 IP/microarray profiles of untreated cells.

## Results

### A method for isolating and identifying miRNAs and mRNAs associated with Ago2

Ago2 is a core component of the RISC complex, associating both with miRNAs and their mRNA targets [21], [24]. Thus, immunopurifying Ago2 under the appropriate conditions might retain associated miRNAs and mRNAs, allowing miRNA targets to be identified. At the outset of the project no effective antibodies were available for immunopurifying Ago2 from mammalian cells. We therefore chose to express a FLAG-tagged Ago2 protein and identify mRNAs and miRNAs that were associated with it.

**Figure 1:**
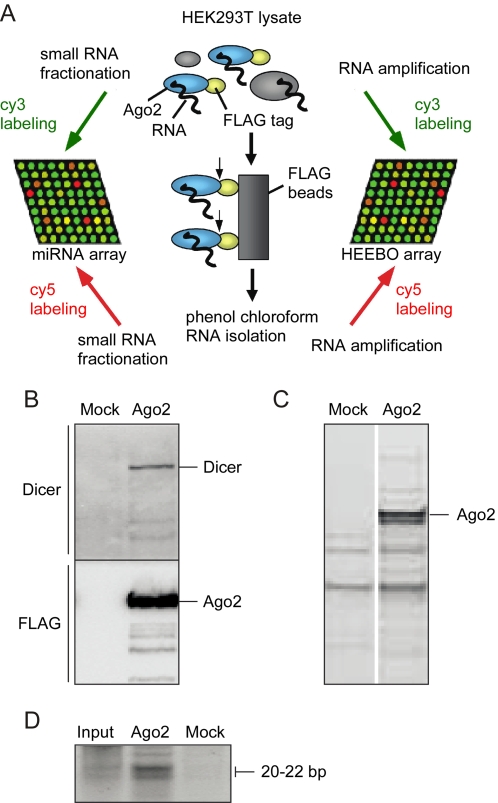
Ago2 association with Dicer and miRNAs. (A) Strategy for the systematic isolation and identification of RNA associated with Ago2. (B) Immunopurification of FLAG protein purified from mock transfected cells (left) and FLAG-Ago2 transfected cells (right). A Dicer antibody (top) and FLAG antibody (bottom) were immuno-reactive with bands corresponding to the predicted molecular weight of Dicer (∼250 kD) and Ago2 (∼90 kD). (C) SYRO ruby protein stain of FLAG immunopurifications from mock transfected cells (left) and FLAG-Ago2 transfected cells (right). (D) SYBR gold nucleic acid stain of small RNAs (20–40 bp) isolated from whole cell lysate (left), FLAG immunopurification from FLAG-Ago2 transfected cells (middle) and FLAG purification from mock transfected cells (right). Brackets outline expected migration of nucleic acids ∼21 base pairs in length.

HEK293T cells were transfected with an N-terminal FLAG-tagged Ago2 construct, allowed to express FLAG-Ago2 for 48 h, and lysed (Figure 1A). Whole cell lysates from transfected cells were mixed with a FLAG antibody resin, resin was then washed, and RNA bound to the resin was recovered by phenol-chloroform extraction. To control for nonspecific association of RNAs with the resin, lysates of mock-transfected cells were subjected to the same affinity purification.

FLAG IPs of FLAG-Ago2 transfected cells were enriched, relative to mock IPs, in the RISC components Ago2 and Dicer (Figure 1B and 1C), in small RNAs (Figure 1D), and in total RNA (5–10 fold; data not shown), consistent with successful purification of the RISC complex and associated RNAs. This enrichment of RNA and Dicer was lost when IPs were performed with a two-fold higher KCl concentration, whereas Ago2 enrichment was not affected (Figure S1).

For microarray analysis, total RNA was isolated from crude lysates, amplified, and labeled with Cy3, and Ago2-associated RNA was obtained from FLAG IPs, amplified, and labeled with Cy5. The labeled RNAs were profiled by comparative hybridization to DNA microarrays printed with the Human Exonic Evidence Based Oligonucleotide (HEEBO) probe set containing ∼45,000 70mer oligonucleotide probes designed to detect transcripts for almost all known protein-encoding genes, alternatively spliced transcripts for hundreds of genes, as well as many annotated non-coding RNAs, mitochondrial-encoded mRNAs, and viral RNAs [61]. Small RNAs enriched by size fractionation were labeled with Cy dyes and hybridized to microarrays containing 21mer probes designed to detect ∼300 known human miRNAs [62].

### Effects of Ago2 overexpression on mRNA and miRNA profiles

A major concern was that the miRNAs and mRNAs associated with overexpressed Ago2 might not be completely representative of those normally associated with endogenous Ago2. Indeed, although cells transfected with the FLAG-Ago2 construct were normal in appearance and size, they grew at only ∼75% the rate of mock-transfected cells, indicating some perturbation induced by the overexpression of FLAG-Ago2.

**Figure 2:**
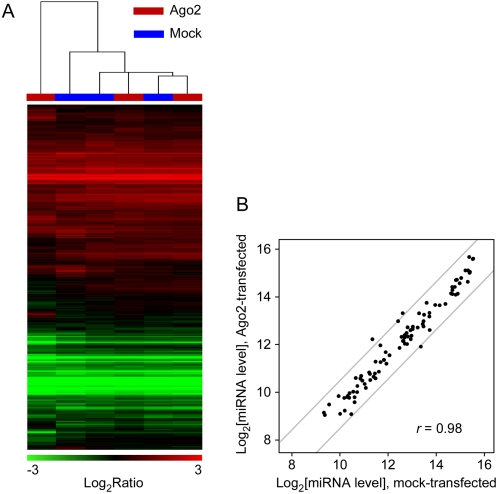
Overexpression of FLAG-Ago2 does not perturb overall mRNA expression or miRNA expression. (A) Unsupervised hierarchical cluster of mRNA levels in HEK293T cells determined relative to a universal reference for RNA from FLAG-Ago2 transfected cells (red) and mock transfected cells (blue). Rows correspond to 12,931 gene elements (representing ∼9,059 genes) and columns represent individual experimental samples (*r_ave_* = 0.92, Pearson correlation between averaged values from each side of the highest node in the dendrogram). (B) Scatter plot of the normalized log_2_ microarray signal intensity of 90 expressed miRNAs from whole cell lysates of mock transfected cells (x-axis) versus the normalized log2 microarray signal intensity from Ago2 transfected cells (y-axis, *r* = 0.98). Values are the averages of 3 experiments. The grey lines delineate the boundary for a two-fold change.

One method for gauging changes in cell physiology is global gene expression profiling, which is a sensitive way of assessing changes in cell state since most physiological responses are associated with changes in mRNA expression [63]. As shown in Figure 2, the mRNA profiles from the Ago2-transfected and mock-transfected samples were very similar (Dataset S1). Hierarchical clustering showed that the Ago2-transfected samples did not segregate away from the mock-transfected samples (Figure 2A), consistent with the hypothesis that Ago2-transfection had little effect on mRNA levels. Using the significance analysis of microarrays (SAM) algorithm [64], we found only one transcript with significant differential expression between Ago2- and mock-transfected samples: a ∼25 fold enrichment of a CMV IRES sequence present in the exogenous FLAG-Ago2 expression transcript. Endogenous Ago2 mRNA levels did not change, as measured by a probe designed to detect the 3′-UTR of endogenous Ago2. Thus, FLAG-Ago2 overexpression had little detectable effect on mRNA profiles.

We also assessed whether FLAG-Ago2 overexpression altered the cells' miRNA profile (Dataset S4). For the 90 miRNAs deemed to be expressed in HEK293T cells, the levels of expression in the presence and absence of FLAG-Ago2 transfection were very similar, with only a few miRNAs registering changes of around two-fold between the two conditions (Figure 2B). Thus FLAG-Ago2 overexpression had little effect on the miRNAs present.

The striking similarity in the mRNA and miRNA profiles for the Ago2 and mock transfected cells suggests that Ago2 overexpression does not substantially alter the global gene expression program at the mRNA level. These results give us confidence that conclusions drawn from our IPs are also applicable to unperturbed HEK293T cells.

### Ago2 immunopurifications contain a representative profile of the cells' miRNAs and a specific subset of their total mRNAs

**Figure 3:**
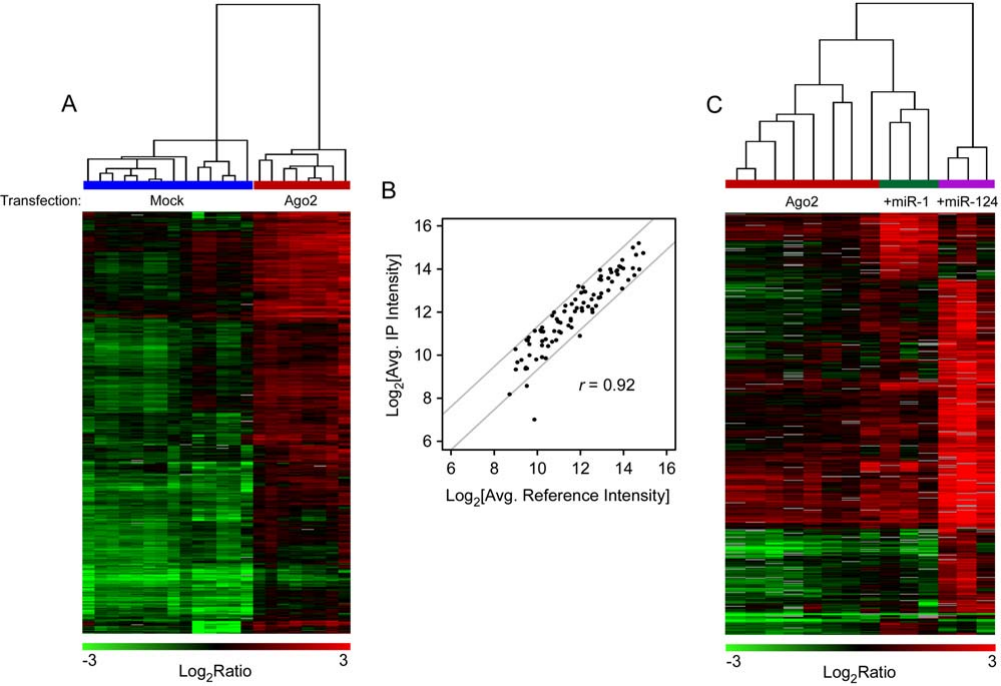
Comparison of mRNA and miRNA specifically associated with Ago2 in the absence or presence of miR-1 or miR-124. (A) Supervised hierarchical cluster of putative Ago2 targets that are enriched over mock (local FDR 1%) from FLAG purifications of FLAG-Ago2 transfected cells (red) and mock transfected cells (blue). Rows correspond to 1,215 gene elements (representing ∼1,083 genes) and columns represent individual experimental samples. There is a high correlation between replicate experiments: *r_ave_* = 0.80 for Ago2 replicates, 0.73 for mock replicates, and −0.070 for all experiments. (B) Scatter plot of the normalized log_2_ microarray signal intensities of 90 miRNAs from whole cell lysate (x-axis) graphed against the normalized log_2_ microarray signal intensities of miRNAs associated with Ago2 (y-axis, *r* = 0.92). Four replicates were performed for each experiment. The grey lines delineate the boundary for a two-fold change. (C) Supervised hierarchical clustering of putative miR-1 and miR-124 targets enriched over Ago2 alone (1% local FDR) from FLAG purifications of FLAG-Ago2 transfected cells alone (red) and FLAG-Ago2 transfected cells with miR-1 (green) or miR-124 (purple). Rows correspond to 667 gene features (representing ∼544 genes) and columns represent individual experimental samples. *r_ave_ = *0.80 for Ago2 replicates, 0.77 for Ago2+miR-1 replicates, 0.90 for Ago2+miR-124 replicates, and 0.43 for all experiments.

HEK293T cells were transfected with FLAG-Ago2, and the FLAG-Ago2 associated miRNAs and mRNAs were isolated, amplified, labeled with Cy dyes, and analyzed by hybridization to HEEBO and miRNA arrays (Dataset S1). The microarray data were then analyzed by SAM. As shown in Figure 3A, 1215 mRNAs were overrepresented in the FLAG IPs from FLAG-Ago2-transfected cells relative to mock-transfected cells at a local false discovery rate (FDR) [65] of 1%. Supervised hierarchical clustering showed that the profiles of mRNAs significantly enriched in 8 FLAG-Ago2-transfections were clearly similar to each other and distinct from 14 mock transfection profiles (Figure 3A), demonstrating the reproducibility of the association of this subset of mRNAs with Ago2. The conservatively-estimated ∼1200 overrepresented mRNAs presumably represent messages being actively regulated by miRNAs under basal conditions. Based upon gene ontology (GO) terms, the Ago2-associated mRNAs were diverse in their functional themes, highlighting the likely importance of RISC and miRNA-mediated regulation in diverse cellular processes.

We also compared the FLAG-Ago2-associated miRNAs to the overall pool of miRNAs obtained from FLAG-Ago2-transfected HEK293T cells (Dataset S4). For the 90 miRNAs judged as being detectably expressed, their representation in the FLAG-Ago2 IPs (Figure 3B, *y*-axis) was found to be proportional to their overall abundance (Figure 3B, *x*-axis), with the exception of a single miRNA (miR-485-5P) that was about 4-fold underrepresented in the FLAG IP. Thus, FLAG-Ago2 IPs contained a fairly representative array of the cells' miRNAs and a select subset of the total mRNAs.

### Systematic identification of mRNAs regulated by miR-1 and miR-124

To identify mRNAs that were recruited to RISC in response to particular miRNAs, we transfected HEK293T cells with FLAG-Ago2 with or without one of two miRNAs not normally expressed in HEK293T cells (miR-1 or miR-124), and assessed how the Ago2-associated mRNA profile was affected (Datasets S2 and S3). We then used SAM to test for mRNAs whose association with Ago2 was significantly higher in the Ago2-plus-miR-transfected cells relative to the Ago2-transfected cells.

The transfection of miR-1 and miR-124 promoted the association of distinct sets of mRNAs with FLAG-Ago2 and presumably RISC. At a stringent 1% local FDR, SAM identified 68 mRNAs specifically recruited by miR-1 and 419 mRNAs specifically recruited by miR-124. Fifty-nine and 388 of these mRNAs, respectively, had RefSeq IDs with 3′-UTR sequences available. Hierarchical clustering of mRNAs based on their association with Ago2 in response to each miRNA revealed a distinct, reproducible target signature for each miRNA (Figure 3C). There was little overlap between the sets of mRNAs; only three mRNAs were targeted to Ago2 by both miR-1 and miR-124. These data provide strong evidence that each of these miRNAs recruits a distinct, reproducible set of mRNAs to FLAG-Ago2-containing RISC complexes.

### Seed matches in the 3′-UTRs of putative miR-1 and miR-124 targets

In principle, the mRNAs specifically associated with Ago2 in cells transfected with miR-1 or miR-124 could have been targeted to Ago2 either directly by the transfected miRNA or by an indirect mechanism; for example, by other miRNAs whose abundance or activity is enhanced indirectly by miR-1 or miR-124. If the miRNA specific Ago2 associated mRNAs consisted predominantly of direct targets, we would expect that many would contain seed matches to the 5′ ends of the respective miRNAs. As an initial approach to this question, we examined what fraction of the high confidence miR-1 and miR-124 targets possessed seed sequences. By the least stringent definition of a seed match – a six-nucleotide match complementary to nucleotides (nt) 2–7 or nt 3–8 in the miRNA – 70% of the miR-1 targets and 75% of the miR-124 targets possessed seed matches, a highly significant (P<10^−6^ and <10^−25^, hypergeometric density distribution) enrichment over would be expected by chance (Table 1, designated ‘6mer’ seed match). For other more stringent definitions of seed matches (7mer: complementarity at nt 2–8 or complementarity at nt 2–7 plus an A at target position 1; 8mer: complementarity at nt 2–8 and an A at target position 1) the percentage of the miR-1 and miR-124 targets with seed matches was lower but still highly significant (Table 1). This indicates that the majority of miR-1 and miR-124 targets are likely to be direct targets of the miRNAs.

**Table 1:** Enrichment of seed match sites to miR-1 and miR-124 in Ago2 IP targets (1% local FDR).

Location	Seed match type	miR-1 (56 IP targets)			miR-124 (388 IP targets)		
		# IP targets with site	% IP targets with site	P-value	# IP targets with site	% IP targets with site	P-value
3′-UTR	6mer	39	70	10^−6^	292	75	10^−25^
	7mer	31	55	10^−11^	205	53	10^−52^
	8mer	17	30	10^−13^	57	15	10^−28^
Coding	7mer	16	29	0.007	128	33	10^−20^
Sequence	7mer/no 3′-UTR 6mer	10	18, 58*	10^−5^	45	9, 47*	10^−15^
5′-UTR	7mer	1	2	0.5	10	3	0.02
	7mer/no 3′-UTR 6mer	1	2, 6*	0.01	6	2, 6*	0.0005

* Percentage relative to the number of targets that do not contain a 3′-UTR 6mer seed match. P-values were calculated from hypergeometric distribution function.

**Figure 4:**
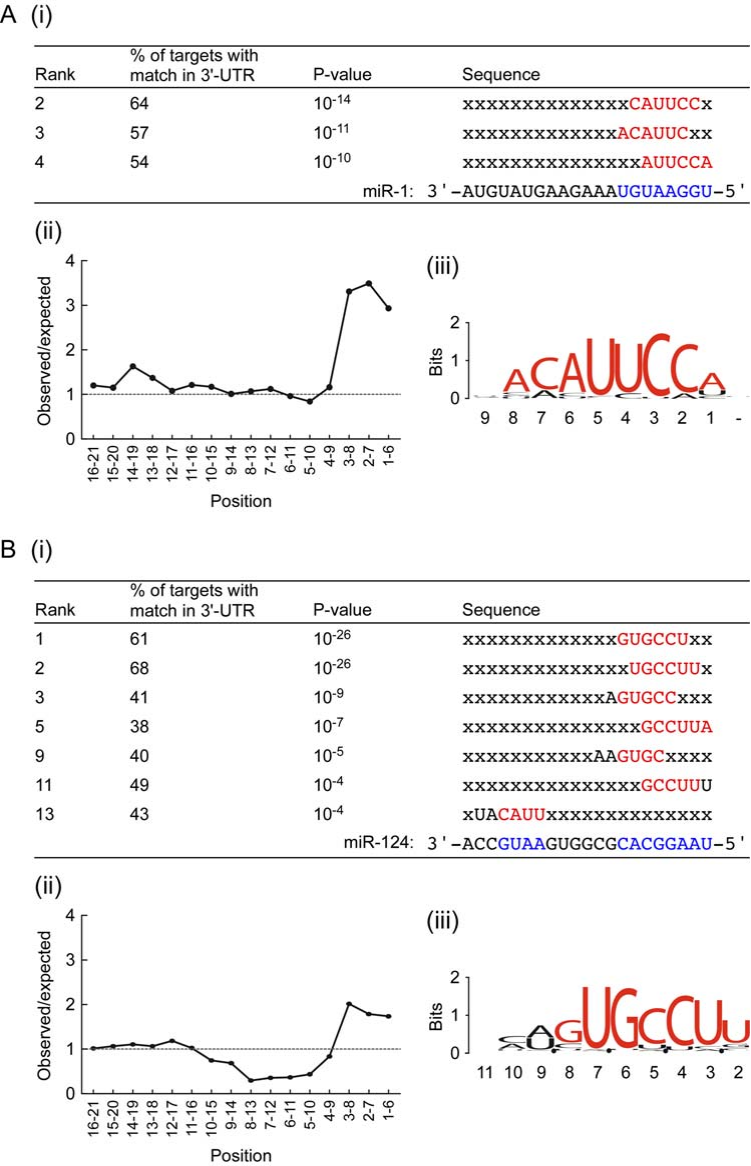
Significantly enriched motifs in 3′-UTRs of mRNAs targeted to Ago2 by miR-1 and miR-124. (A) Analysis of mRNAs associated with Ago2 from cells transfected with FLAG-Ago2 and miR-1 relative to cells transfected with Ago2 alone (1% local FDR). (i) Enrichment of hexamers in 3′-UTRs of miR-1 IP targets compared to 3′-UTRs of all mRNAs passing array filters. Shown are hexamers with at least four contiguous Watson-Crick base pairs to miRNA with a p-value cut-off of 0.001 (binomial test with bonferroni correction). Rank by p-value relative to all 4096 hexamers. Bases in red can form Watson-Crick base pairs with miR-1. (ii) Moving plot of observed/expected ratios of hexamers complementary to miR-1. Frequencies calculated as in (i). (iii) 10mer motif returned by MEME motif finder using 3′-UTR sequences from the miR-1 high confidence target set. For each position in the motif, the combined height of the bases represents the information content at that position, whereas the relative heights of the individual bases represent the frequency of that base at that position. Bases in red can form Watson-Crick base pairs with miR-1. Numbers underneath the logo correspond with miRNA 5′-position, with 1 being the 5′-most miRNA nucleotide. (B) Same as in (A), except for mRNAs associated with Ago2 from cells transfected with FLAG-Ago2 and miR-124 relative to cells transfected with Ago2 alone.

A second approach to the same question was to ask which 6mers were most highly overrepresented in the 3′-UTRs of the high confidence miR-1 and miR-124 targets. For miR-1, the most highly overrepresented 6mer was UUUUUU. This low complexity sequence is often speciously enriched in small sample sizes because of its frequent occurrence in 3′-UTRs, and is likely not specifically associated with miR-1. The next three most highly overrepresented 6mers were overlapping sequences with perfect complementarity to positions 1–8 in miR-1 (Figure 4Ai). We also calculated the frequency with which a perfect match to each of the 16 6mers in miR-1 was found in the high confidence miR-1 targets. As shown in Figure 4Aii, the three most 5′ 6mers were highly overrepresented. Similarly, the multiple expectation maximization for motif elicitation (MEME) motif discovery algorithm identified a 10 nt motif sequence from the high confidence miR-1 targets with perfect complementarity to positions 1–8 in miR-1 (Figure 4Aiii) [66].

Analogous results were found for miR-124, although in this case there was some enrichment for base pairing near the 3′ end of the miRNA as well as base pairing near the 5′ end (Figure 4Bi and Bii). These results are consistent with previous reports demonstrating no preference for base-pairing immediately adjacent to the seed match, but some base-pairing with miRNA positions 13–18 [50], [51]. The 10 nt motif returned by MEME included a 7 nt stretch with perfect complementarity to positions 2–8 in miR-124.

We also looked for overrepresentation of the nt 2–7 seed matches for all 90 of the miRNAs we deemed as being expressed in HEK293T cells in the putative miR-1 and miR-124 targets. None were found to significantly overrepresented; the response appeared to be specific.

Taken together, these data indicate that most of the high confidence targets of miR-1 and miR-124 are likely to be direct targets. In addition, the same sequence criteria inferred from previous studies for the recognition of mRNA targets by miRNAs [30], [31], [48]–[50], especially the importance of sequences complementary to positions 1–8 in the miRNA, emerged independently from analysis of the mRNAs overrepresented in FLAG-Ago2 IPs. These results provide an important validation of the IP method.

### Relationship between overrepresentation in Ago2 immunopurifications and underrepresentation in the bulk mRNA pool

miRNAs appear to regulate gene expression by effects on mRNA abundance or translation or both [11], [32]–[47]. Therefore, previous studies focusing on changes in mRNA levels alone potentially miss many targets. We hypothesized that the IP method could capture all direct mRNA targets regardless of functional outcome. To test this, we measured mRNA levels from cells transfected with the respective miRNAs in parallel to the IP experiments (Datasets S2 and S3). We found 0 and 145 mRNAs significantly decreased in presence of miR-1 and miR-124, respectively, at a 1% local FDR threshold. Thus significantly more putative miRNA targets, 56 and 388 respectively, were identified by miRNA-specific enrichment Ago2 IPs than from the miRNA-specific changes in mRNA levels.

**Figure 5:**
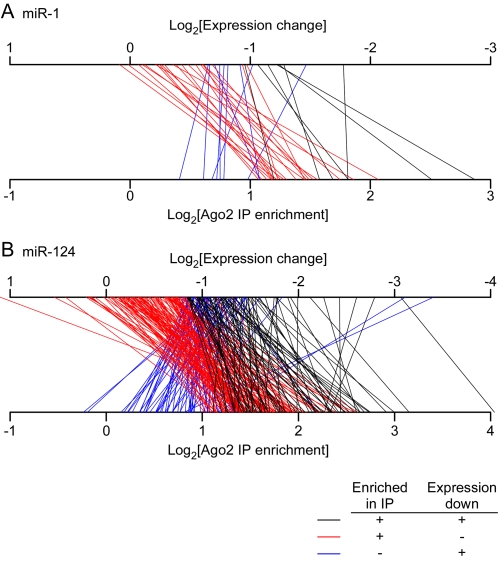
Relationship between overrepresentation in Ago2 IP and changes in mRNA levels due to miR-1 and miR-124. (A) Lines connect the log_2_ of the average Ago2 IP enrichment value (bottom axis) to the log_2_ of the average mRNA expression change (top axis) for three groups of mRNAs from miR-1 experiments. This analysis contains only mRNAs with 7mer seed matches in their 3′-UTRs. Black lines correspond to mRNAs that were Ago2 IP targets (1% local FDR) and decreased significantly at the mRNA level (10% local FDR); seven mRNAs are in this group. Red lines correspond to mRNAs that were Ago2 IP targets but did not decrease significantly at the mRNA level; nevertheless, 20 of 22 mRNAs in this group are downregulated (log_2_ change<0) at the mRNA level (P<10^−5^, one-way binomial test). Blue lines correspond to mRNAs that decreased significantly at the mRNA level, but were not Ago2 IP targets; all 9 mRNAs in this group are overrepresented (log_2_ enrichment>0) in Ago2 IPs (P = 0.0006). (B) Same as in (A) except for mRNAs from miR-124 experiments. 82 mRNAs are in the black group. 109/121 mRNAs in the red group are downregulated at the mRNA level (P<10^−15^); and 63/65 mRNAs in the blue group are overrepresented in Ago2 IPs (P<10^−15^).

To further explore the relationship between Ago2 IP enrichment and mRNA expression change, we relaxed the stringency for the SAM analysis of miRNA-induced decreases in mRNA abundance to a 10% local FDR threshold (16 for miR-1 and 255 for miR-124), and mapped the values from each assay onto two axes (Figure 5). We broke the data into three color coded classes: mRNAs that are overrepresented in Ago2 IPs and decrease in mRNA level (Figure 5, black lines); mRNAs that are overrepresented in Ago2 IPs but do not significantly decrease in mRNA level (Figure 5, red lines); and mRNAs that are not overrepresented in Ago2 IPs, but decrease significantly at the mRNA level (Figure 5, blue lines). To enrich for the highest confidence targets, we focused on mRNAs with a 7mer seed match in their 3′-UTRs.

Only a minority of the targets that were significantly overrepresented in the Ago2 IPs were also significantly decreased in their mRNA levels: 24% of the miR-1 IP targets and 40% of miR-124 IP targets (Figure 5, black lines). These targets are represented by lines that run from the positive side of the Ago2 IP axis to the negative side of the expression axis (Figure 5, black lines). On average the mRNA levels of these miR-1 and miR-124 targets decreased 58% and 61%, respectively. The majority of the targets overrepresented in Ago2 IPs did not show significant decreases (10% local FDR) in their overall mRNA levels (Figure 5, red lines). Nevertheless, approximately 90% of these IP targets did show some decrease in their expression levels (Figure 5, red lines), with average decreases of 24% and 25%, respectively. Thus, most of the mRNAs overrepresented in Ago2 IPs did exhibit a modest decrease in their mRNA levels. The mRNA targets whose levels decreased only modestly may be regulated primarily at the translation level or miRNAs may play only small modulatory roles in the expression of the proteins these mRNAs encode.

Conversely, 44% and 56% of mRNAs that decreased significantly in response to miR-1 and miR-124 transfection, respectively, were not significantly overrepresented (1% local FDR) in the Ago2 IPs (Figure 5, blue lines). However, almost all of these (9/9 miR-1 targets and 63/65 miR-124 targets) were enriched to some extent in the Ago2 IPs to some extent (Figure 5, blue lines). This trend argues that most of these mRNAs are actually miR-1 and miR-124 targets.

### Relationship between size and number of seed matches and overrepresentation in Ago2 immunopurifications

Bartel and co-workers [50] previously reported that mRNAs with long seed match sites (e.g. 8mer matches) were more likely to change in abundance in response miRNA transfection than those with shorter seed match sites, and that mRNAs with two 7mer seed match sites were more likely to show changes than those with one. We therefore asked whether the same relationships would hold based on overrepresentation of mRNAs in Ago2 IPs. As shown in Figure S2, this was indeed the case. For both miR-1 and miR-124, mRNAs with a single 8mer seed match site were overrepresented in the Ago2 IPs relative to those with single 7mer seed match sites, and those with single 7mer seed match sites were overrepresented relative those with single 6mer seed match sites (Figure S2A and B). Likewise, mRNAs with two or more 7mer seed match sites were overrepresented in the Ago2 IPs relative to those with one (Figure S2C and D). The same relationships were found in the changes of mRNA levels in response to miR-1 and miR-124 (data not shown). These findings corroborate and extend those of Grimson et al. [50] and further validate the Ago2 IP method.

### Analysis of putative target mRNAs that lack 3′-UTR seed matches

Interestingly, a large minority, 30% and 25%, of high confidence miR-1 and miR-124 targets identified by Ago2 IP do not contain a 6mer seed match in their 3′-UTRs. Several studies have provided evidence that seed matches in the coding sequence and 5′-UTR can also confer regulation by a miRNA, as judged by mRNA expression data, Ago2 IPs in Drosophila, phylogenetic conservation analyses and reporter studies [6], [43], [50], [53]–[55]. We therefore checked for enrichment of 7mer seed matches in the coding sequences of miR-1 and miR-124 Ago2 IP targets. As reported in Table 1, 29% and 33% of Ago2 IP targets contained coding sequence 7mer seed match sites for miR-1 and miR-124 respectively (P = 0.007 and 10^−20^, hypergeometric distribution). Further, 59% and 47% of miR-1 and miR-124 targets that lacked any 3′-UTR seed matches contained 7mer seed matches in their coding sequences (P<10^−5^ and 10^−15^; Table 1). 5′-UTR 7mer seed matches were also significantly overrepresented (P = 0.001 and 0.0005 for miR-1 and miR-124, respectively; Table 1).

**Figure 6:**
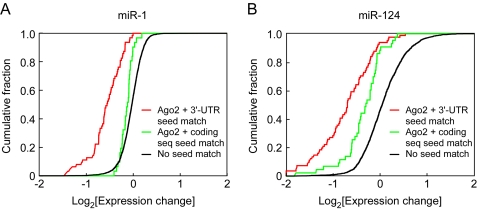
Comparison of expression changes of mRNAs containing seed matches in 3′-UTRs and coding sequences of miR-1 and miR-124 Ago2 IP targets. (A) Cumulative distribution of the change in mRNA levels following transfection with FLAG-Ago2 and miR-1 compared to FLAG-Ago2 alone. This analysis included Ago2 IP targets with 3′-UTR 7mer seed matches, but no coding sequence 6mer seed matches (21, red), Ago2 IP targets with coding sequence 7mer seed matches, but no 3′-UTR 6mer seed matches (10, green), and mRNAs that did not contain 3′-UTR or coding sequence 6mer seed matches (2893, black). Changes in mRNA levels of Ago2 IP targets with 3′-UTR 7mer seed matches were greater than those for Ago2 IP targets with coding sequence 7mer seed matches (P = 0.0004), which were in turn greater than those for mRNAs without any 6mer seed matches in the 3′-UTR or coding sequence (P = 0.006). (B) Same as in (A) except for mRNAs associated with FLAG-Ago2 upon transfection with miR-124. There were 81 Ago2 IP targets with 3′-UTR 7mer seed matches but no 6mer coding sequence seed matches (red), 43 Ago2 IP targets with coding sequence 7mer seed matches but no 6mer 3′-UTR seed matches (green), and 1877 mRNAs with no 6mer seed matches in their 3′-UTR or coding sequence. Changes in mRNA levels of Ago2 IP targets with 3′-UTR 7mer seed matches were greater than the changes for Ago2 IP targets with coding sequence 7mer seed matches (P = 0.0005), which in turn were greater than the changes for mRNAs without any 6mer seed matches in the 3′-UTR or coding sequence (P<10^−8^).

We next set out to assess the effectiveness of 3′-UTR seed matches versus coding sequence seed matches in the Ago2 IP targets, as assessed by effects on mRNA expression (Figure 6A and B). Seed matches in the 5′-UTR were not included in this analysis because of their small numbers. We considered two subsets the miR-1 and miR-124 targets: those mRNAs that possessed a 7mer seed match in the 3′-UTR but no 6mer seed match in their coding sequence (Figure 6, red curves) and those that possessed a 7mer seed match in their coding sequence but no 6mer seed match in their 3′-UTR (Figure 6, green curves). As a comparison group we examined all mRNAs (not just those whose levels in the Ago2 IPs changed after miRNA transfection) that contained no 6mer seed match (Figure 6, black curves). We then plotted the cumulative distributions for each subset as a function of the miRNA-induced change in expression level (Figure 6). If one type of seed match was highly effective at causing mRNA expression decreases, we would expect the cumulative distribution of that subset to be shifted to the left with respect to the black curve. That was the case for the coding sequence seed matches, although the shift was modest (Figure 6A and B, green) (P<0.006 and 10^−8^ for miR-1 and miR-124, respectively). The 3′-UTR seed matches shifted further to the left (Figure 6A and B, red) (P = 0.0004 and 0.00005 for miR-1 and miR-124 respectively). Thus, coding sequence seed matches appeared to be effective in decreasing mRNA levels, but 3′-UTR seed matches were more effective. These conclusions agree well with previous studies based on other assays and approaches.

As a further test of the significance of coding sequence seed matches, we compared how well conserved they were compared to 3′-UTR seed matches. Of the high confidence miR-1 and miR-124 targets with 7mer 3′-UTR seed matches, 41% and 47% of the mRNAs contained seed matches that were perfectly conserved across mouse, rat and dog. Of the high confidence targets with 7mer coding sequence seed matches, 25% and 35% of the mRNAs contained conserved seed matches across the same species. We also compared whether high confidence targets were more likely to contain conserved seed matches than were non-targets for both coding sequence and 3′-UTR seed matches. For 7mer 3′-UTR seed matches, the conservation rate was higher in Ago2 IP targets than in non-targets (41% vs. 22% for miR-1 and 47% vs. 45% for miR-124; P = 0.005 and 0.3). For 7mer coding sequence seed matches, the conservation rates were very similar for targets versus non-targets (25% for targets vs. 29% for non-targets for miR-1; 35% for targets vs. 36% for non-targets for miR-124; P = 0.4 and 0.6). These comparisons strongly suggest that 3′-UTR seed matches are more important than coding sequence seed matches for the regulation of mRNA levels [6], [43], [50], [53]–[55].

### Estimation of the number of mRNAs regulated by miR-1 and miR-124

**Figure 7:**
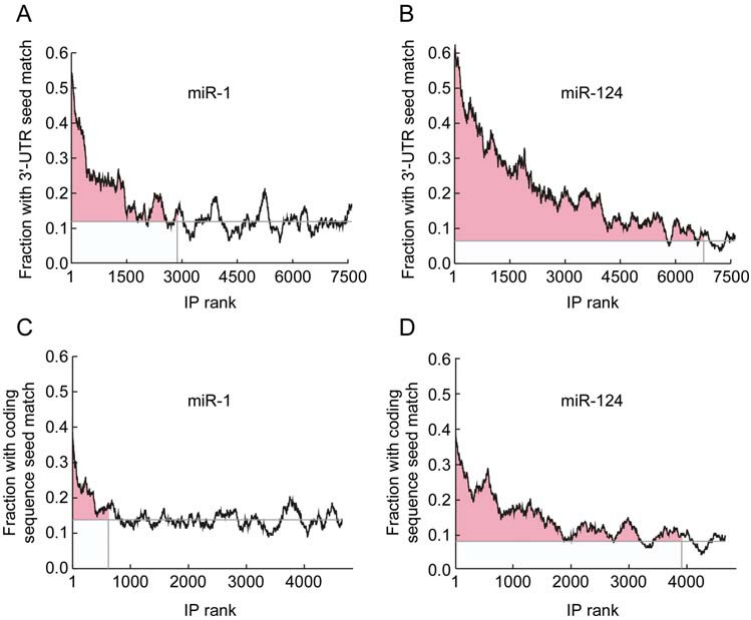
Estimation of the number of miR-1 and miR-124 targets. (A) Moving average plot (window size of 200) of the fraction of mRNAs with 7mer 3′-UTR seed matches to miR-1. mRNAs were ranked by their SAM enrichment in Ago2 IPs in the presence of miR-1 compared to Ago2 alone, with 1 corresponding to the most enriched mRNA. To determine the point at which the curve first rose above the background level of 7mer seed matches, we first calculated the slope of each least-squares-fit regression line between the right-hand end of the distribution and every point to left of it. The point at which the curve first rose above the background level of 7mer seed matches was determined as the point at which the slope was first negative (vertical grey line). The fraction of mRNAs containing 7mer seed matches to the right of the vertical grey line was considered to be the background level of 7mer seed matches (horizontal grey line). To estimate the total number of targets (pink shaded region), the number of mRNAs to the left of the vertical grey line (3071 of 7805) was multiplied by the fraction of mRNAs to the left of the vertical line containing 7mer 3′-UTR seed matches (0.23) minus the fraction of mRNAs to the right of the vertical line containing 7mer seed matches (0.12). This results in an estimate of 325 targets. (B) Same as in (A), but for miR-124. 6393 of 7817 mRNAs were to the left of the vertical grey line. The fraction of mRNAs with 7mer 3′-UTR seed matches to the left of the grey vertical line was 0.21, while the fraction of mRNAs with 7mer seed matches to the right of the grey vertical lines was 0.07. This results in an estimate of 1000 targets. (C) Same as in (A), except moving average plots of the fraction of mRNAs with 7mer coding sequence seed matches to miR-1. mRNAs with 6mer 3′-UTR seed matches were removed, leaving 4855 mRNAs. 820 mRNAs were to the left of the vertical grey line. The fraction of mRNAs with 7mer seed matches to the left and right of the grey vertical line was 0.24 and 0.14 respectively. This results in an estimate of 83 targets. (D) Same as in (C), but for miR-124. 3312 of 3916 mRNAs were to the left of the vertical grey line. The fraction of mRNAs with 7mer seed matches to the left and right of the grey vertical line was 0.15 and 0.08 respectively. This results in an estimate of 236 targets.

Several thousand human mRNAs have seed match sites to either miR-1 or miR-124, but only a small fraction of these were identified with high confidence as actual regulatory targets by our gene expression profiling and IP experiments. We estimated a lower bound of 68 targets for miR-1 and 419 for miR-124, based on the stringent 1% local FDR criterion. Several prediction algorithms and reporter assays along with our analysis in Figure 5 suggest that the total number of targets is significantly higher [6], [31], [49]–[52], [58], [59]. We therefore took an alternative statistical approach to this question. We ranked the 7805 (for miR-1-transfected cells) and 7817 (for miR-124-transfected cells) well-measured mRNAs with RefSeq IDs from most enriched to least enriched in Ago2 IPs (Figure 7A and B, *x*-axes). Then for groups of 200 sequences, we calculated running averages of the fraction of mRNAs with 7mer seed matches in their 3′-UTRs. As expected, the fraction was high for the most enriched mRNAs and low for the least (Figure 7A and B). We then estimated where the curve first significantly rose above the background frequency of 7mer seed matches. To accomplish this we calculated the slope between the right-hand end of the distribution and every point to the left of it. We took the cutoff to be where the slope of this line first became negative (Figure 7A and B, vertical gray line). We then calculated the estimated number of targets as:
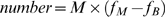
where *M* is the number of mRNAs to the left of the cutoff, *f_M_* is the fraction of those mRNAs with 7mer seed matches, and *f_B_* is the fraction of the mRNAs to the right of the cutoff with 7mer seed matches. This method gives estimates of 325 and 1000 mRNAs recruited to Ago2 by 3′-UTR seed matches to miR-1 and miR-124, respectively (Figure 7A,B). Using 6mer seed matches rather than 7mers yielded similar estimates, 293 and 1232 respectively (data not shown). Applying the same logic to mRNAs with 7mer seed matches in their coding sequences but no 6mer 3′-UTR seed matches gives estimates of 83 miR-1 and 236 miR-124 targets recruited to Ago2 exclusively through miRNA targeting of the coding sequence (Figure 7C and D). Using 6mer seed matches again yielded similar estimates (50 and 253; data not shown). These data provide direct empirical evidence that miR-1 and miR-124 have hundreds of direct mRNA targets. These miRNAs are highly connected hubs in the network of RNA regulation.

### Functions of the high confidence miR-1 and miR-124 targets

To determine if miR-1 and miR-124 selectively bind mRNAs that share common biological functions we searched for enrichment of GO terms in the miRNAs target sets identified through Ago2 immunopurification. There is modest enrichment of several GO categories for each miRNA target set: for example, the miR-124 set is enriched for mRNAs encoding proteins localized to the membrane (P = 0.002) or that bind GTP (P = 0.009), and the miR-1 set is enriched for mRNAs involved mRNA metabolism (P = 0.006) and cell motility (P = 0.015). We also tested the distribution of ∼430 curated gene sets in the IP enrichments as a whole. Using curated gene sets from gene set enrichment analysis [67] rather than GO terms, we found that no gene sets were significantly enriched at a corrected P-value threshold of 0.05.

### Using Ago2 immunopurification enrichment and mRNA expression changes to assess computational target prediction methods

Our empirical data on miR-1 and miR-124 targets allow us to assess computational methods for the prediction of miRNA targets. We examined five methods: TargetScan 4.0, which looks for seed matches in appropriate sequence contexts [50]; TargetScan 3.0 and PicTar, which look for seed matches conserved among human, dog, mouse, rat, and chicken mRNAs [31], [52]; PITA, which makes use of seed matches and predicted target accessibility [58]; and MiRanda, which looks at sequence complementarity and conservation among human, mouse and rat mRNAs [49], [51]. The performance of these methods was assessed by cumulative distribution plots, with either microarray expression data or Ago2 IP enrichment data on the *x*-axis (Figure S3). The more successful the method, the further its cumulative distribution curve should shift to the left (for expression data) or the right (for Ago2 IP enrichment). As shown in Figure S3, TargetScan 4.0 performed best for predicting both miR-1 targets and miR-124 targets (blue curves). TargetScan 3.0 and PicTar were next best (magenta and orange curves), followed by PITA and Miranda (green and gray curves). None of the computational methods performed as well as an expression data-plus-seed match criterion for predicting Ago2 IP-enriched targets (Figure S3A and B, red curves). Likewise, none of the computational methods performed as well as an Ago2 IP-enrichment-plus-seed match criterion for predicting targets identified by the expression data (Figure S3C and D, red curves). Thus, while TargetScan 4.0 performed particularly well, the two empirical methods (expression data and Ago2 IP enrichment) were superior. This indicates that some information important for miRNA target recognition is still missing from the prediction algorithms.

## Discussion

### A direct assay to identify targets of specific miRNAs

Much of what we know about miRNA targeting has been inferred indirectly from effects on mRNA levels, from phylogenetic conservation of recognition sites, and, on a smaller scale, from effects on levels of the encoded proteins. Such studies have provided a foundation for our understanding of miRNA regulation of gene expression, yet crucial information directly linking these effects to miRNA pathways has been missing. We would like to know what mRNAs are recruited to RISC by each miRNA and are thus acted upon by miRNA-mediated regulation.

A simple method employing immunoaffinity isolation of Ago2, a core component of RISC, identifies mRNAs recruited to RISC by specific miRNAs. Knowledge of these mRNAs provides a direct and critical point of reference for understanding the molecular mechanism and logic of mRNA target specificity and for comprehensive investigation of the functional consequences of miRNA-induced interactions.

The selective association of specific mRNAs with Ago2 in response to specific miRNAs is *prima facie* evidence for their miRNA-mediated recruitment to Ago2/RISC. The enrichment of cognate seed matches in computationally predicted favorable contexts and the correlation between specific miRNA-dependent Ago2-IP enrichment and changes in mRNA levels are strong evidence that the assay reflects a direct and functional interaction between the transfected miRNA and the Ago2 IP-enriched mRNAs. Although the introduction of exogenous FLAG-Ago2 could have altered the normal specificity of these interactions, the negligible effect of exogenous FLAG-Ago2 on global patterns of expression of either mRNAs or miRNAs argue against a major distortion of normal regulation and suggest that the interactions we observed are generally faithful representations of native interactions.

The evolutionary conservation of many of the seed matches to miR-1 and miR-124 in the 3′-UTRs of the mRNAs identified as specific targets in our assay lends further credence to the biological relevance of the interactions. Transfection of miR-1 or miR-124 into these cells greatly increased the cellular levels of these miRNAs (which are undetectable in untransfected cells), but the resulting concentrations appeared to be well below those of the most highly expressed endogenous miRNAs, based on qRT-PCR experiments (data not shown). The lack of enrichment (or underenrichment) of seed matches to other miRNAs in the mRNAs recruited to Ago2-RISC after transfection of miR-1 or miR-124 further implies that perturbation of endogenous miRNAs and miRNA targets was not a significant factor in the Ago2-enriched mRNAs.

The cells we used in this study were chosen for their experimental tractability; they were not an optimal model for studies of the regulatory roles of miR-1 or miR-124. The Ago2 IP procedure used herein should, however, be widely applicable to other cells or even whole organisms, in which mRNAs identified as targets, by either overexpressing or blocking a specific miRNA, can be related to specific biological consequences.

Although there were significant changes in the levels of many of the mRNAs recruited to Ago2/RISC in the presence of specific miRNAs, there were also many associated mRNAs that were only slightly altered in expression level. The ability to identify mRNA targets directly, without relying on a change in their levels in response to perturbation of a specific miRNA, makes it possible to systematically investigate other possible miRNA-directed effects on their expression, including, for example, effects on subcellular localization or translation.

### Functional insights into miRNA targeting and regulation

The strong correlation between miRNA-specific association with Ago2 and decreases in mRNA levels for mRNA targets with 3′-UTR seed matches suggests that the strength or properties of a miRNA's association with a potential target mRNA has an important role in regulating their degradation. Most high confidence mRNA targets with 3′-UTR seed matches were regulated to some degree at the level of mRNA abundance, albeit weakly in most cases.

Comparing the magnitude of the changes in mRNA abundance to direct measurements of the efficiency with which each mRNA is recruited to RISC revealed a quantitative relationship between the recruitment efficiency and consequences for expression. Moreover, the link between size, number of seed matches and recruitment efficiency suggests an evolutionary mechanism for quantitative tuning of the regulatory response of each mRNA to a miRNA. A continuous scale of regulation, tuning affinity, or context of a site that allows an increase in existing regulation is easier than evolving a functional site *de novo*.

### Insights into miRNA-based regulation from recent, related publications

While this paper was in preparation, four papers describing similar strategies to identify mRNAs associated with RISC and specific miRNAs were published [53], [68]–[70]. We briefly review these results, highlighting the similarities that strengthen common conclusions and differences that may be instructive with respect to methodologies and biological mechanisms.

Easow *et al.* [53] immunopurified affinity-tagged Ago1 from *Drosophila melanogaster* S2 cells and identified associated mRNAs via microarray hybridization. The authors found 89 mRNAs specifically associated with Ago1. 3′-UTR seed matches to some highly expressed miRNAs were overrepresented in the target set. The authors also found some enrichment of coding sequence seed matches to highly expressed miRNAs in the Ago1 targets. The efficacy of coding seed match sites was tested for two mRNAs lacking seed matches in their 3′-UTR by cloning the coding sequences in-frame with a luciferase reporter; mutation of these seed match sites led to a ∼25% increase in the expression of luciferase. The authors also created two exogenous seed matches to a highly expressed miRNA in the coding sequence of luciferase by mutating silent codon positions. Sequences with these seed matches were also introduced into the SV40 3′-UTR downstream of a luciferase reporter. Addition of the seed match sites to both regions led to decreased luciferase levels, but the repression was more pronounced when the sites were in the 3′-UTR. 108 mRNAs were differentially underenriched in Ago1 IPs of embryos with a mutation in miR-1 compared to Ago1 IPs from lysates of embryos with the wild-type miR-1 gene. The regulatory effect of miR-1 on potential targets was gauged by comparing the luciferase levels of reporter genes containing 3′-UTRs of 11 of the 32 potential miR-1 targets in the presence or absence of miR-1. Luciferase activity was reduced in the presence of miR-1 for all 11 constructs, but to different extents, varying from ∼5–60%. In all cases, the decreases in luciferase activity were greater than the decreases in mRNA levels, consistent with substantial translational regulation, as suggested by numerous studies.

Zhang *et al.* [70] immunopurified GFP-tagged AIN-1 and AIN-2, which they found to be Ago-associated proteins, from *Caenorhabditis elegans* whole animals and identified associated miRNAs by sequencing and associated mRNAs by DNA microarray hybridization. The authors found approximately 3000 (15% of all known *C. elegans* genes) mRNAs associated with either AIN-1 or AIN-2, including many known and predicted miRNA targets.

Beitzinger *et al.* [68] immunopurified Ago1 and Ago2 from HEK293T cells and identified some associated mRNAs by sequencing. About 600 clones derived from RNAs associated with Ago1 or Ago2 were sequenced. Nonspecific interactions with the resin or antibody were not controlled for by comparison to “mock” immunopurification. Instead, clones recovered once were classified as nonspecific and clones recovered multiple times were counted as direct targets. Using this criterion, only 82 unique Ago1 and 28 unique Ago2 targets were found, with 15 targets in common. The large number of single hit clones indicates that the results represent a non-exhaustive list of Ago2 targets. Thus, it is not surprising that the Ago2-associated mRNAs identified in this study do not significantly overlap with our findings.

Karginov et al. [69] applied an Ago2 immunopurification strategy similar to ours to identify Aog2-associated mRNAs and miRNAs in HEK293T cells. The authors found over one thousand mRNAs specifically associated with Ago2. Ago2 IP enrichment was positively correlated with the presence of 3′-UTR seed match sites to highly expressed miRNAs. Following transfection of miR-124, 370 mRNAs were specifically recruited to Ago2. About half of the putative targets contained 7merm-8 seed matches to miR-124 in their 3′-UTRs. The mRNA levels of many of the putative miR-124 targets were significantly decreased in response to the presence of miR-124. The 3′-UTRs from 21 of 30 mRNAs that were miR-124 IP targets but did not change significantly at the mRNA level that were incorporated into luciferase probes lead to significant miR-124 dependent decreases in protein expression. Of the 370 mRNAs for which Karginov et al. reported a miR-124 dependent association with Ago2, 238 had unique RefSeq IDs and were detected on our arrays. Forty-nine percent of these mRNAs were also classified as miR-124 targets in our experiments at a 1% local FDR threshold, 59% were enriched at a less stringent 10% local FDR cut-off, and 95% were more enriched than the median IP enrichment of all mRNAs. Our findings are thus in broad general agreement, and provide further validation of the approach and individual insights into miRNA-based regulation.

There were also findings that were unique to our study. Enrichment of mRNAs containing coding sequence and 5′-UTR seed match sites was not observed in the Karginov et al. study. The discrepancies may be related to differences in the IP procedures. Karginov et al. washed immunopurified beads with 650 mM NaCl, whereas our immunopurifications and washes were performed with 150 mM KCl. In our hands, washing the Ago2 immunopurified beads with 300 mM KCl resulted in loss of enrichment of total RNA and Dicer, whereas Ago2 enrichment was not affected (SFigure 1). We did not analyze the pool of mRNA that remained bound to Ago2 following this more stringent wash. It is possible that the stringent wash employed in Karginov et al. disrupted relatively labile mRNA:RISC interactions, including those in coding sequences and 5′-UTRs. On the other hand, it is possible that coding sequence and 5′-UTR interactions identified in our assay were “created” by association with Ago2 post-lysis. Another reason for the discrepancy could be differences in the growth rate of the cells at the time of lysis. The global ribosome occupancy at the time of lysis in the cells used in our experiments was quite low because the cells reached high confluency during the 48 hours between transfection and cell-lysis. It is possible that under conditions in which there is more translation, coding sequence and 5′-UTR sites are relatively less occupied compared to 3′-UTR sites, because these miRNA-mRNA interactions are disrupted by ribosomes. The Karginov et al. study employed similar growth conditions, and also prepared extracts 48 hours after transfections. Regardless of the causes for the discrepancies, the significant decrease in abundance of mRNAs containing coding sequence sites suggests that these are biologically relevant miRNA targets.

## Materials and Methods

### Plasmids and oligonucleotides

CMV-FLAG-Ago2 plasmid was provided by G. Meister and T. Tuschl [21].

miR-1 siRNA:
sense: 5′UGGAAUGUAAAGAAGUAUGUA3′
antisense: 5′CAUACUUCUUUACAUUCAAUA3′
miR-124 siRNA:
sense: 5′UAAGGCACGCGGUGAAUGCCA3′
antisense: 5′GCAUUCACCGCGUGCCUUAAU3′

### Cell culture and transfection

HEK293T cells were obtained from ATCC (Cat #CRL-11268) and grown in Dulbecco's modified Eagle's medium (Invitrogen) with 10% fetal bovine serum (Invitrogen) and supplemented with 100 U/ml penicillin, 100 mg/ml streptomycin, and additional 4 mM glutamine (Invitrogen) at 37°C and 5% CO_2_. Transfections of HEK293T cells were carried out with calcium phosphate. Cells were plated in 10 cm dishes 12–16 hrs prior to transfection at 30% confluency. For 1 mL transfection mixtures (1/10 volume of growth media) 61 µl of 2 M CaCl_2_ and 10 µg of Ago2 plasmid DNA were diluted into 500 µl of nuclease free H_2_O (Invitrogen) and added slowly to 500 µl of 2× HBS (50 mM Hepes, 280 mM NaCl, 1.5 mM Na_2_HPO_4_) pH 7.1. After ∼1 minute, the mixture was added to a 10 cm plate at a medium pace. Transfections with the miR-1 and miR-124 oligonucleotides were performed analogously by diluting a 40 µM stock to 5 nM in the 500 µl nucleic acid mixture along with the plasmid DNA.

### Imunoaffinity purification and RNA isolation

For each purification, 400 µl of 4°C lysis buffer (150 mM KCl, 25 mM Tris-HCl pH 7.4, 5 mM EDTA, 0.5% Nonidet P-40, 5 mM DTT, 1 mM PMSF, 50 µM chymostatin, 50 µM leupeptin, 50 µM pepstatin, 0.5 µM aprotinin, 100 U/ml SUPERase•In (Ambion Cat #AM2694) was added to ten 10 cm plates after washing 1× in PBS 48 hrs post-transfection. After 30 min at 4°C, the plates were scraped and the lysates combined and spun at 4°C for 30 minutes at 14,000 RPM in a microcentrifuge. The supernatant was then collected and filtered through a 0.45 µm syringe filter. The lysate was then mixed with 300 µl of FLAG resin (Sigma Cat #A2220), which was equilibrated by washing 2× with lysis buffer with 10× volume. The beads were incubated with the lysate for 4 hrs at 4°C and washed 2× with 10× volume of lysis buffer for 5 minutes. Five percent of the beads were frozen for SDS PAGE analysis after the second wash. RNA was extracted directly from the remaining beads with 25∶24∶1 phenol∶chloroform∶isoamyl alcohol (Invitrogen Cat#15593-031). Trace amounts of phenol were removed by chloroform extraction and RNA was precipitated using sodium acetate with Glyco-Blue (Ambion Cat# AM9516) as a carrier. RNA pellets were resuspended in 25 µl of RNase free water and stored at −80°C. Small RNA samples for PAGE detection were isolated using a modified protocol for RNA isolation using Invitrogen's Micro-to-Midi kit (Invitrogen Cat#12183-18) [71]. Small RNA for microarray analysis was fractionated using the FLASH-PAGE system (Ambion Cat#AM13100) as per vendors' instructions.

### Western blots, Sypro staining, and nucleic acid PAGE

Resin saved from each immunoaffinity purification were resuspended in water and diluted to 1× sample buffer and 1× reducing buffer (Biorad Cat#161-0791, and 161-0792) and heated at 95°C for 3 min. Each sample was then divided and one-half was loaded onto either a 4–12% criterion XT gel (BioRad Cat# 345-126) for protein staining with sypro ruby (Invitrogen Cat #S-12000) or onto a 3–8% criterion XT gel for western blotting (BioRad Cat# 345-0129). For the SYPRO ruby staining, gels were treated as per the vendor's instruction immediately after electrophoresis. For western analysis, each gel was transferred onto polyvinylidene fluoride membrane (Immobilon Cat# IPVH08100) and probed with FLAG m2 antibody (Sigma Cat# F-1804) and a polyclonal Dicer antibody generated by rabbits inoculated with a peptide corresponding with the N-terminus of Dicer: EILRKYKPYERQQFESVC (Quality Controlled Biochemicals). Small RNA was detected using 15% urea TBE criterion gels (Biorad Cat# 345-0055) and Syber Gold (Invitrogen Cat# S-1149) as per the vendor's instructions. RNA (0.5–1 µg) was loaded in each lane.

### Microarray production and pre-hybridization processing

Detailed methods for microarray experiments are available at the Brown lab website (http://cmgm.stanford.edu/pbrown/protocols/index.html). HEEBO oligonucleotide microarrays and miRNA microarrays were produced by Stanford Functional Genomic Facility. The HEEBO microarrays contain ∼45,000 70-mer oligonucleotide probes, representing ∼30,000 unique genes. A detailed description of this probe set can be found at (http://microarray.org/sfgf/heebo.do) [61]. The miRNA arrays (Ambion miRNA Bioarrays version 2) [62] contained probes for 668 human, mouse and rat miRNAs. Each probe was printed in duplicate.

RNA from immunopurification experiments was hybridized to microarrays printed on aminosilane-coated glass (Schott Nexterion A). Prior to hybridization, the oligonucleotides were cross-linked to the aminosilane-coated surface with 65 mJ of UV irradiation. Slides were then incubated in a 500 ml solution containing 5× SSC (1× SSC = 150 mM NaCl, 15 mM sodium citrate, pH 7.0), 1% w/v Blocking Reagent (Roche Cat# 1109617001), and 0.1% SDS for 35 minutes at 65°C. Slides were washed twice for 1 min each in glass chambers containing 400 ml water, dunked in a glass jar containing 400 ml 95% ethanol for 15 seconds, then dried by centrifugation. Slides were used the same day.

mRNA expression experiments and miRNA experiments were performed with microarrays printed on epoxysilane-coated glass (Schott Nexterion E). Prior to hybridization, slides were first incubated in a humidity chamber (Sigma Cat# H6644) containing 0.5× SSC for 30 min at room temperature. Slides were snap-dried at 70–80°C on an inverted heat block. The free epoxysilane groups were blocked by incubation with 1M Tris-HCl pH 9.0, 100 mM ethanolamine (Sigma Cat# E9508), and 0.1% SDS for 20 minutes at 50°C. Slides were washed twice for 1 min each with 400 ml water, and then dried by centrifugation. Slides were used the same day.

### Sample preparation, hybridization and washing

For HEEBO microarray experiments, poly-adenylated RNAs were amplified in the presence of aminoallyl-UTP with Amino Allyl MessageAmp II aRNA kit (Ambion Cat# 1753). For expression experiments, universal reference RNA was used as an internal standard to enable reliable comparison of relative transcript levels in multiple samples (Stratagene Cat# 740000). Amplified RNA (3–10 µg) was fluorescently labeled with NHS-monoester Cy5 or Cy3 (GE HealthSciences Cat# RPN5661). Dye-labeled RNA was fragmented (Ambion cat# 8740), then diluted into in a 50 µl solution containing 3× SSC, 25 mM Hepes-NaOH, pH 7.0, 20 µg human Cot-1 DNA (Invitrogen Cat# 15279011), 20 µg poly(A) RNA (Sigma cat # P4303), 25 ug yeast tRNA (Invitrogen Cat # 15401029), and 0.3% SDS. The sample was incubated at 70°C for 5 minutes, spun at 14,000 rpm for 10 minutes in a microcentrifuge, then hybridized at 65°C for 12–16 hours. For immunopurification experiments, microarrays were hybridized inside sealed chambers in a water bath using the M-series lifterslip to contain the probe on the microarray (Erie Scientific Cat # 22x60I-M-5522). For mRNA expression experiments, microarrays were hybridized using the MAUI hybridization system (BioMicro), which promotes active mixing during hybridization.

Following hybridization, microarrays were washed in a series of four solutions containing 400 ml of 2× SSC with 0.05% SDS, 2× SSC, 1× SSC, and 0.2× SSC, respectively. The first wash was performed for 5 minutes at 65°C. The subsequent washes were performed at room temperatures for 2 minutes each. Following the last wash the microarrays were dried by centrifugation in a low-ozone environment (<5 ppb) to prevent destruction of Cy dyes [72]. Once dry, the microarrays were kept in a low-ozone environment during storage and scanning (see http://cmgm.stanford.edu/pbrown/protocols/index.html).

Small RNAs for miRNA microarrays were labeled using the *mir*Vana labeling kit (Ambion Cat# Am1562) and samples were prepared, hybridized and washed according to manufacturer's instructions.

### Scanning and data processing

Microarrays were scanned using either AxonScanner 4200 or 400oB (Molecular Devices). PMT levels were auto-adjusted to achieve 0.1–0.25% pixel saturation. Each element was located and analyzed using GenePix Pro 5.0 (Molecular Devices). These data were submitted to the Stanford Microarray Database for further analysis (http://smd.stanford.edu/cgi-bin/publication/viewPublication.pl?pub_no=685) [73]. Data were filtered to exclude elements that did not have a regression correlation of ≥0.6 between Cy5 and Cy3 signal over the pixels compromising the array element of and intensity/background ratio of ≥2.5 in at least one channel, for 60% of the arrays. For cluster and SAM (Fig 2) analysis of Ago2+/−miR-1/124 IPs versus mock IPs, measurements corresponding to oligonucleotides that map to the same entrezID were treated separately and the data were globally normalized per array, such that the median log_2_ ratio was 0 after normalization. For all analysis after Figure 3, measurements corresponding to oligonucleotides that map to the same entrezID were averaged and the data were globally normalized per array, such that the median log_2_ ratio was 0 after normalization. To control for variation among groups of experiments performed at different times, each group was normalized by subtracting the median log_2_ ratio for each gene across the experiments in a group from the log_2_ ratio of the gene in each experiment. The groups are labeled in the supplementary information.

miRNA microarray experiments were normalized by subtracting the median value of average Cy3 and Cy5 signal intensities of negative control spots from the average Cy3 and Cy5 signal of each experimental measurement. Normalized Cy3 and Cy5 signal intensities from replicate experiments were normalized and log_2_ transformed (measurements with negative values were changed to a value of 1). The distribution of the log_2_ signal intensities was nearly bimodal; miRNAs with signal intensity greater than the value at the trough of the distribution were considered to be expressed.

The microarray data have been submitted to Gene Expression Omnibus (GEO) (www.ncbi.nlm.nih.gov/geo/) under the accession number GSE11082.

### Microarray analyses

Hierarchical clustering was performed with Cluster 3.0 [74] and visualized with Java TreeView 1.0.12 [75].

For SAM, unpaired two-class t-tests were performed with default settings (R-package samr). FDRs were generated from up to 1000 permutations of batch normalized (see above) data.

### Sequence data

For each entrezID, the RefSeq sequence with the longest 3′-UTR was used. In cases where there were multiple RefSeqs with the same 3′-UTR length, the one that was alpha-numerically first was used. RefSeq 3′-UTR, coding, and 5′-UTR sequences were retrieved from UCSC genome browser (hg18). Seed match sites in these sequences were identified with Perl scripts. miR-1 seed matches: 6mer_n2–7 “CAUUCC”, 6mer_n3–8 “ACAUUC”, 7mer-m8 “ACAUUCC”, 7mer-A1 “CAUUCCA”, 8mer “GUGCCUUA”. miR-124 seed matches: 6mer_n2–7 “UGCCUU”, 6mer_n3–8 “GUGCCU”, 7mer-m8 “GUGCCUU”, 7mer-A1 “UGCCUUA”, 8mer “GUGCCUUA”.

### Conservation of seed match sites

For each RefSeq, the 28-way multiple sequence alignments files for the 3′-UTR or coding sequences were retrieved from UCSC genome table browser. The human, dog (canFam2), mouse (mm8), and rat (rn4) sequences were extracted and multiple sequence alignments files corresponding to the same RefSeq were stitched together with Galaxy. Sites with 7mer-m8 or 7mer-A1 matches present in all three species within 20 positions of the human seed match site were considered to be conserved.

### Sequence analyses

Enrichment of hexamers in 3′-UTRs of miR-1 and miR-124 targets relative to nontargets was performed on the Regulatory Sequence Analysis Tools website. P-values were calculated with binomial distribution function and corrected for multiple hypothesis testing using the bonferroni method.

To identify sequence motifs associated with enrichment in immunopurifications subsequent to miR-1 and miR-124 transfection, the MEME method was used [66]. MEME 3.0.14 was downloaded from the MEME website and run with default settings, except searching the forward strand only with zoops model.

### miRNA target Predictions

Predictions for Targetscan 4.0 were downloaded on June 12, 2007. Context scores for each miRNA site in each RefSeq sequence were summed to get the cumulative context score for that miRNA. Predictions for human PITA3/15 flank were downloaded on November 4, 2007. For both TargetScan 4.0 and PITA, miR-506/124-2 predictions were used for miR-124, because of changes in annotated sequences in Sanger miRNA database. These miRNAs share the same 5′end as the miR-124 sequence used in this study, but have different 3′ends. Predictions for TargetScan 3.0 were retrieved on November 2, 2006. Predictions for Pictar 5-way and Miranda were retrieved on March 2, 2007. Miranda predictions used Ensemble IDs, which were mapped to RefSeqs using UCSC genome table browser.

### Gene ontology and gene-set analyses

Enrichment of GO terms in miR-1, miR-124, and Ago2 target sets was identified with Expression Analysis Systematic Explorer [76]. Enrichment of gene sets was performed with Gene Set Enrichment Analysis [67].

## Supporting Information

Figure S1 Disassociation of Dicer from Ago2 IPs in 300 mM KCL. Western blot with Dicer antibody on protein associated with an Ago2 IP from cells lysed in 150 mM KCl (left), after washing once with 300 mM KCL (middle), and after washing a second time with a 300 mM KCl concentration (right).
(0.25 MB TIF)

Figure S2 The length and number of 3′-UTR seed match sites to miR-1 and miR-124 correlates with enrichment in Ago2 IPs. (A) Cumulative distributions of Ago2 IP enrichment due to the presence of miR-1 of mRNAs containing single 6–8mer 3′-UTR seed match sites. The IP enrichment of mRNAs containing different 6–8mer sites was as follows: 8mer (144, red)>7mer-m8 (257, green, P = 0.005, one-sided Mann–Whitney test)>7mer-A1 (375, blue, P = 0.0005)>6mer_n-7 (magenta, 567, P = 0.005)∼6mer_n3–8 (orange, 654, P = 0.3)∼no seed match (black, 4855, P = 0.2). (B) Same as in (A), except for miR-124. The IP enrichment of mRNAs containing different 6–8mer sites was as follows: 8mer (78, red)>7mer-m8 (329, green, P<10-4)>7mer-A1 (283, blue, P<10-4)>6mer_n-7 (magenta, 845, P<10-9)∼6mer_n3–8 (orange, 624, P = 0.09)>no seed match (black, 3916, P<10-4). (C) Cumulative distributions of Ago2 IP enrichment due the presence of miR-1 of mRNAs containing multiple 7mer 3′-UTR seed match sites (61, red), single 7mer 3′-UTR seed match sites (632, green), and no 3′-UTR seed match sites (black). mRNAs containing multiple miR-1 7mer 3′-UTR seed match sites were significantly more enriched than mRNAs containing single 7mer seed match sites (P = 0.03). (D) Same as in (C), except for miR-124. mRNAs containing multiple 7mer 3′-UTR seed match sites (81) were significantly more enriched than mRNAs containing single 7mer seed match sites (612, P = 0.01).
(0.47 MB TIF)

Figure S3 Using Ago2 IP enrichment and mRNA expression changes to assess computational target prediction methods. (A) Cumulative distributions of Ago2 IP enrichment in cells transfected with miR-1 relative to cells transfected with FLAG-Ago2 alone of several sets of mRNAs predicted to be targeted by miR-1. The 100 mRNAs containing 7mer 3′-UTR seed matches to miR-1 whose levels were most significantly downregulated due to the presence of miR-1 were the most enriched group (red). The 100 mRNAs whose levels were most significantly downregulated due to the presence of miR-1 irrespective of seed match sites were the next most enriched group in Ago2 IPs (dark green). The 100 mRNAs containing the lowest TargetScan 4.0 “cumulative context score” were the next most enriched group (blue). TargetScan 3.0 (274, magenta) and PicTar 5way predictions (97, orange) were the next most enriched groups. The 100 mRNAs containing the most favorable 3′-UTRs for miR-1 binding according to PITA 3/15 flank (green), miRanda predictions (113, grey) and mRNAs containing 7mer 3′-UTR seed matches were the next most enriched groups (1245, cyan). mRNAs containing no 6mer 3′-UTR seed matches were the least enriched group (4765, black). (B) Same as in (A) except for miR-124. 573 mRNAs were TargetScan 3.0 predictions; 128 mRNAs were PicTar 5way predictions, 202 mRNAs were miRanda predictions, 1500 mRNAs contained 7mer 3′-UTR seed matches, and 3820 mRNAs did not contain a 6mer 3′-UTR seed match. (C) Cumulative distributions of changes in mRNA levels in cells transfected with miR-1 relative to cells transfected with FLAG-Ago2 alone of several sets of mRNAs predicted to be targeted by miR-1. The 100 mRNAs containing 7mer 3′-UTR seed matches to miR-1 that were most enriched in Ago2 IPs due to the presence of miR-1 was the most underenriched group (red). The 100 mRNAs that were most enriched in Ago2 IPs due to the presence of miR-1 irrespective of seed match sites was the next group (dark green). The 100 mRNAs containing the lowest TargetScan 4.0 “cumulative context score” was the next most underenriched group (blue). TargetScan 3.0 (magenta) and PicTar 5way predictions (orange) were the next most underenriched groups. The 100 mRNAs containing the most favorable 3′-UTRs for miR-1 binding according to PITA 3/15 flank (green), miRanda predictions (grey) and mRNAs containing 7mer 3′-UTR seed matches were the next most underenriched groups (cyan). mRNAs containing no 6mer 3′-UTR seed match was the least underenriched group (black). (D) Same as in (C) except for miR-124.
(0.74 MB TIF)

Dataset S1 Ago2 versus mock, IP and expression HEEBO microarray data
(10.08 MB XLS)

Dataset S2 Ago2/miR-1 versus Ago2, IP and expression HEEBO microarray data
(14.85 MB XLS)

Dataset S3 Ago2/miR-124 versus Ago2, IP and expression HEEBO microarray data
(14.55 MB XLS)

Dataset S4 miRNA microarray data
(14.55 MB XLS)
