# Correction: Systematic Identification of mRNAs Recruited to Argonaute 2 by Specific microRNAs and Corresponding Changes in Transcript Abundance

**DOI:** 10.1371/annotation/c8902b4c-30fc-42e7-b506-fc756a3cdd4e

**Published:** 2008-11-14

**Authors:** David G. Hendrickson, Daniel J. Hogan, Daniel Herschlag, James E. Ferrell, Patrick O. Brown

The file for Dataset S4 is a duplicate of Dataset S3. Please view the correct file for Dataset S4 here:
