# Supplementary figures and images for: Systematic Identification of mRNAs Recruited to Argonaute 2 by Specific microRNAs and Corresponding Changes in Transcript Abundance
Source: PLoS One. 2008 May 7;3(5):e2126. doi: 10.1371/journal.pone.0002126 (PMC2330160; doi:10.1371/journal.pone.0002126)

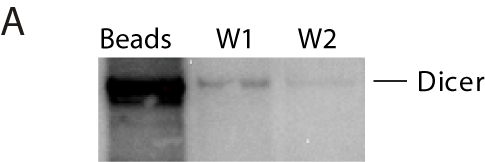

Supplement: Figure S1 — Disassociation of Dicer from Ago2 IPs in 300 mM KCL. Western blot with Dicer antibody on protein associated with an Ago2 IP from cells lysed in 150 mM KCl (left), after washing once with 300 mM KCL (middle), and after washing a second time with a 300 mM KCl concentration (right). (0.25 MB TIF) [file pone.0002126.s001.tif]

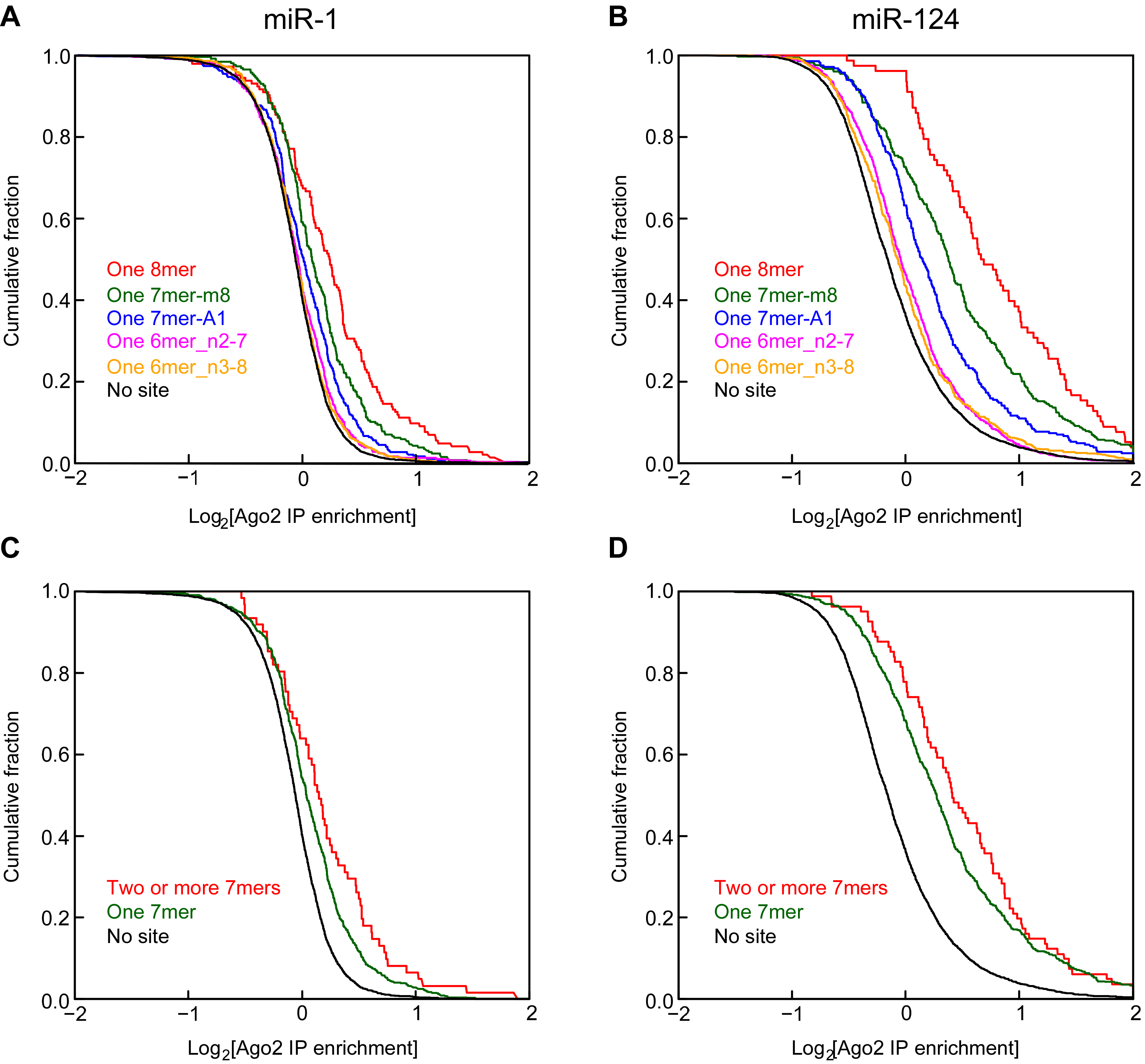

Supplement: Figure S2 — The length and number of 3′-UTR seed match sites to miR-1 and miR-124 correlates with enrichment in Ago2 IPs. (A) Cumulative distributions of Ago2 IP enrichment due to the presence of miR-1 of mRNAs containing single 6–8mer 3′-UTR seed match sites. The IP enrichment of mRNAs containing different 6–8mer sites was as follows: 8mer (144, red)>7mer-m8 (257, green, P = 0.005, one-sided Mann–Whitney test)>7mer-A1 (375, blue, P = 0.0005)>6mer_n-7 (magenta, 567, P = 0.005)∼6mer_n3–8 (orange, 654, P = 0.3)∼no seed match (black, 4855, P = 0.2). (B) Same as in (A), except for miR-124. The IP enrichment of mRNAs containing different 6–8mer sites was as follows: 8mer (78, red)>7mer-m8 (329, green, P<10-4)>7mer-A1 (283, blue, P<10-4)>6mer_n-7 (magenta, 845, P<10-9)∼6mer_n3–8 (orange, 624, P = 0.09)>no seed match (black, 3916, P<10-4). (C) Cumulative distributions of Ago2 IP enrichment due the presence of miR-1 of mRNAs containing multiple 7mer 3′-UTR seed match sites (61, red), single 7mer 3′-UTR seed match sites (632, green), and no 3′-UTR seed match sites (black). mRNAs containing multiple miR-1 7mer 3′-UTR seed match sites were significantly more enriched than mRNAs containing single 7mer seed match sites (P = 0.03). (D) Same as in (C), except for miR-124. mRNAs containing multiple 7mer 3′-UTR seed match sites (81) were significantly more enriched than mRNAs containing single 7mer seed match sites (612, P = 0.01). (0.47 MB TIF) [file pone.0002126.s002.tif]

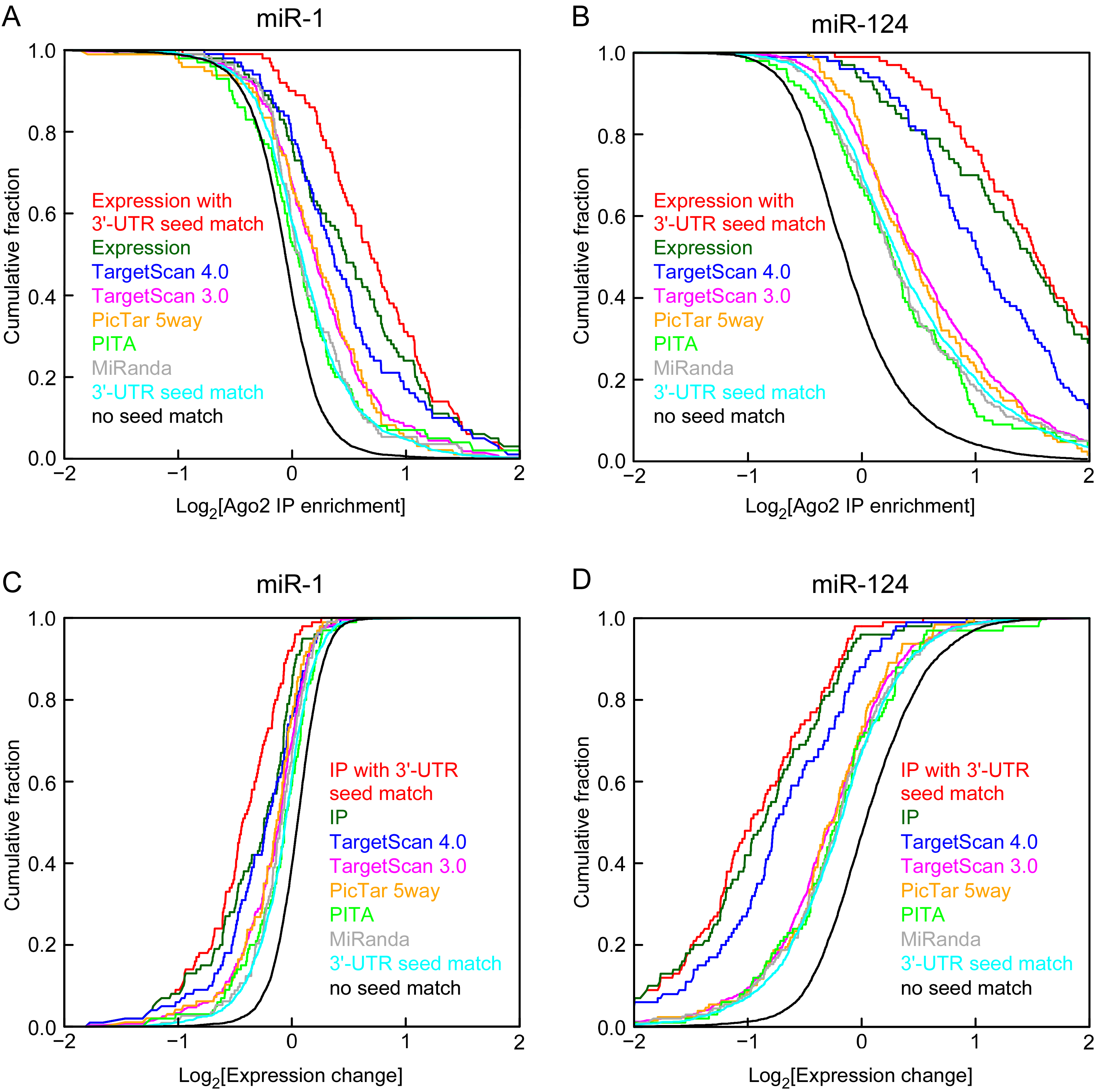

Supplement: Figure S3 — Using Ago2 IP enrichment and mRNA expression changes to assess computational target prediction methods. (A) Cumulative distributions of Ago2 IP enrichment in cells transfected with miR-1 relative to cells transfected with FLAG-Ago2 alone of several sets of mRNAs predicted to be targeted by miR-1. The 100 mRNAs containing 7mer 3′-UTR seed matches to miR-1 whose levels were most significantly downregulated due to the presence of miR-1 were the most enriched group (red). The 100 mRNAs whose levels were most significantly downregulated due to the presence of miR-1 irrespective of seed match sites were the next most enriched group in Ago2 IPs (dark green). The 100 mRNAs containing the lowest TargetScan 4.0 “cumulative context score” were the next most enriched group (blue). TargetScan 3.0 (274, magenta) and PicTar 5way predictions (97, orange) were the next most enriched groups. The 100 mRNAs containing the most favorable 3′-UTRs for miR-1 binding according to PITA 3/15 flank (green), miRanda predictions (113, grey) and mRNAs containing 7mer 3′-UTR seed matches were the next most enriched groups (1245, cyan). mRNAs containing no 6mer 3′-UTR seed matches were the least enriched group (4765, black). (B) Same as in (A) except for miR-124. 573 mRNAs were TargetScan 3.0 predictions; 128 mRNAs were PicTar 5way predictions, 202 mRNAs were miRanda predictions, 1500 mRNAs contained 7mer 3′-UTR seed matches, and 3820 mRNAs did not contain a 6mer 3′-UTR seed match. (C) Cumulative distributions of changes in mRNA levels in cells transfected with miR-1 relative to cells transfected with FLAG-Ago2 alone of several sets of mRNAs predicted to be targeted by miR-1. The 100 mRNAs containing 7mer 3′-UTR seed matches to miR-1 that were most enriched in Ago2 IPs due to the presence of miR-1 was the most underenriched group (red). The 100 mRNAs that were most enriched in Ago2 IPs due to the presence of miR-1 irrespective of seed match sites was the next group (dark green). The 100 mRNAs conta [file pone.0002126.s003.tif]
